# Evaluation of a Low Cost Hand Held Unit with GNSS Raw Data Capability and Comparison with an Android Smartphone

**DOI:** 10.3390/s18124185

**Published:** 2018-11-29

**Authors:** Gérard Lachapelle, Paul Gratton, Jamie Horrelt, Erica Lemieux, Ali Broumandan

**Affiliations:** PLAN Group, Schulich School of Engineering, University of Calgary, Calgary, AB T2N 1N4, Canada; paul.gratton@ucalgary.ca (P.G.); jamie.horrelt@ucalgary.ca (J.H.); erica.lemieux1@ucalgary.ca (E.L.); a.broumandan@gmail.com (A.B.)

**Keywords:** raw GNSS data, Android smartphone, hand held receivers

## Abstract

A newly available portable unit with GNSS raw data recording capability is assessed to determine static and kinematic position accuracy in various environments. This unit is the GPSMap 66, introduced by Garmin in early September. It is all-weather and robust for field use, and comes with a helix antenna. The high sensitivity chipset is capable of acquiring and tracking signals in highly attenuated environments. It can track single frequency GPS, GPS + GLONASS or GPS + Galileo and record code, Doppler and carrier phase data every second in the RINEX format. The evaluation presented herein focusses on GPS and Galileo. Static and kinematic test results obtained under a wide range of realistic field conditions are reported. Differential GNSS methods and Precise Point Positioning (PPP) are used to assess absolute position accuracy in ITRF coordinates, which is sufficiently close to the GPS and Galileo reference frame for the current purpose. Under low multipath conditions, measurements are found to be sufficiently accurate to provide single epoch, bias free position accuracy of a few metres. Accuracy is a function of signal attenuation and multipath conditions. The use of an external geodetic antenna significantly reduces measurement noise and multipath in high multipath environments. Carrier phase measurements, available more or less continuously under open sky conditions, significantly improve performance in differential mode. Accuracy in vehicular mode using code and carrier phase differential RTK solution is at the level of a few to several dm. Tests were conducted in parallel with a Huawei P10 Android 8.0 smartphone. The code measurement noise of this unit was found to be significantly higher than that of the GPSMap 66, a major reason being its lower performance PIFA antenna; carrier phase was only available for short time intervals, significantly degrading differential position accuracy performance.

## 1. Introduction

Android smartphones that can record raw GNSS pseudorange (and most recently carrier phase) measurements have been available since late 2016 and initial results have shown promising potential for positioning (e.g., [1,2]). Since a main purpose of a smartphone is communication, the GNSS capability comes at limited additional expense. The data can be processed in real-time or post-mission. In the latter case, online precise point positioning (PPP) services are available to process data collected in static or kinematic mode. Open source software such as RTKLib (rtklib.org) is also available for single point and differential processing.

**Figure 1:**
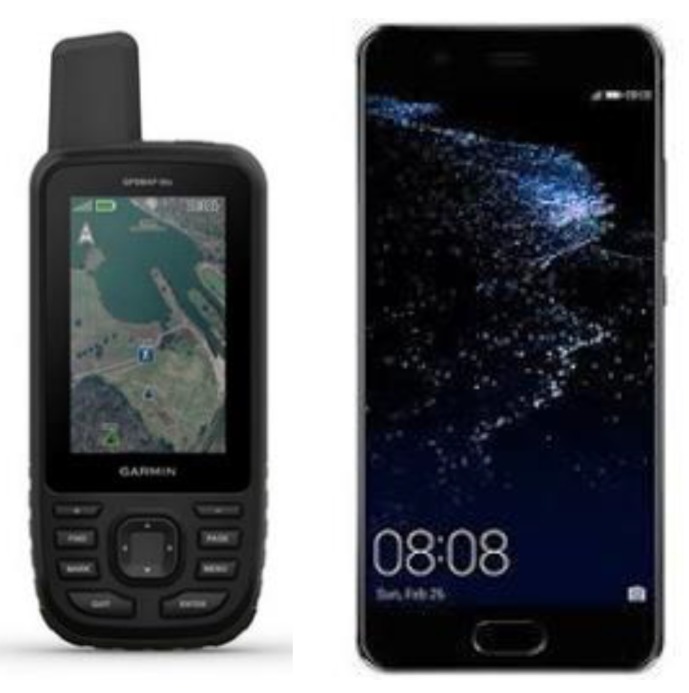
Garmin GPSMap 66 and Huawei P10 units tested.

Low cost portable and wearable units with high sensitivity GNSS chips, similar to those available in smartphones, have been available for years for a variety of applications. It was only a matter of time before manufacturers offered raw data availability to potentially enhance performance for applications where needed. These units are robust and all weather. Garmin (Kansas City, MO, USA) has recently announced its GPSMap 66 with the optional recording of observation files in the RINEX format. The unit, shown in Figure 1 (left) is equipped with a quadrifilar helix antenna and a high sensitivity chip capable of tracking and recording either GPS, GPS + Galileo or GPS + GLONASS raw data. The latter consists of raw code, Doppler, carrier phase and carrier-to-noise ratio (C/N_0_) measurements every second, and associated time tags. The evaluation reported herein focusses on GPS and Galileo. Performance is compared to that of a P10 cellphone (Huawei, Shenzhen, China) running under the Android 8 operating system phone when possible. The P10 is also shown in Figure 1. The unit can track most GNSS signals. The P10 is the first generation of Android phones than can record continuous raw carrier phase data, subject to the limitations of the operating system.

## 2. Test Procedure

Two GPSMap 66 units (labeled blue and green) were used to confirm reproducibility of results during testing. The test procedures and analysis methodologies used for the equipment evaluation are common to evaluate GNSS equipment performance, well documented in the literature and require no further description.

Testing conditions ranged from ideal horizon-to-horizon open sky to indoors with signal attenuation of over 25 dB. The following tests are described herein:

(1) Single point static positioning in a high specular multipath environment occurring on a building roof top. In this case, a high grade geodetic GNSS external antenna was also tested with the units under evaluation to demonstrate antenna characteristic importance in such an environment.
(2) Single point and differential static GNSS in a relatively low multipath environment occurring in open areas.
(3) Vehicular testing on relatively open roads with units mounted on the vehicle roof to allow access to satellite lines-of-sight.
(4) Indoor signal analysis with signal attenuation of over 25 dB.
(5) Extreme urban canyon signal analysis testing.

A point precisely (within 1–2 cm) known in ITRF 2014 (basically equivalent to WGS84, given the accuracy required for the present assessment) was used for some of the single point static tests and served as base to establish coordinates of other points in precise differential GNSS mode when needed. For differential static and vehicular tests, a high-end R10 geodetic receiver (Trimble, Sunnyvale, CA, USA) was used to derive reference positions with accuracy of 10 cm or better. Hourly NASE broadcast ephemerides were used to generate the GPSMap 66 and P10 results.

Measurements were processed using RTKLib in forward mode for both single point and differential positioning. No filtering was used when processing data in single point mode in order to assess raw code data performance, with the understanding that this would not be done in practice. Some GPS measurements were also reprocessed in PPP mode using the Natural Resources Canada (NRCan) latest CSRS-PPP online software calculator (V 1.05 11216) which provides geodetic coordinates in ITRF 2014; the software processes L1/L2 or L1 only, both with carrier phase measurements.

**Figure 2:**
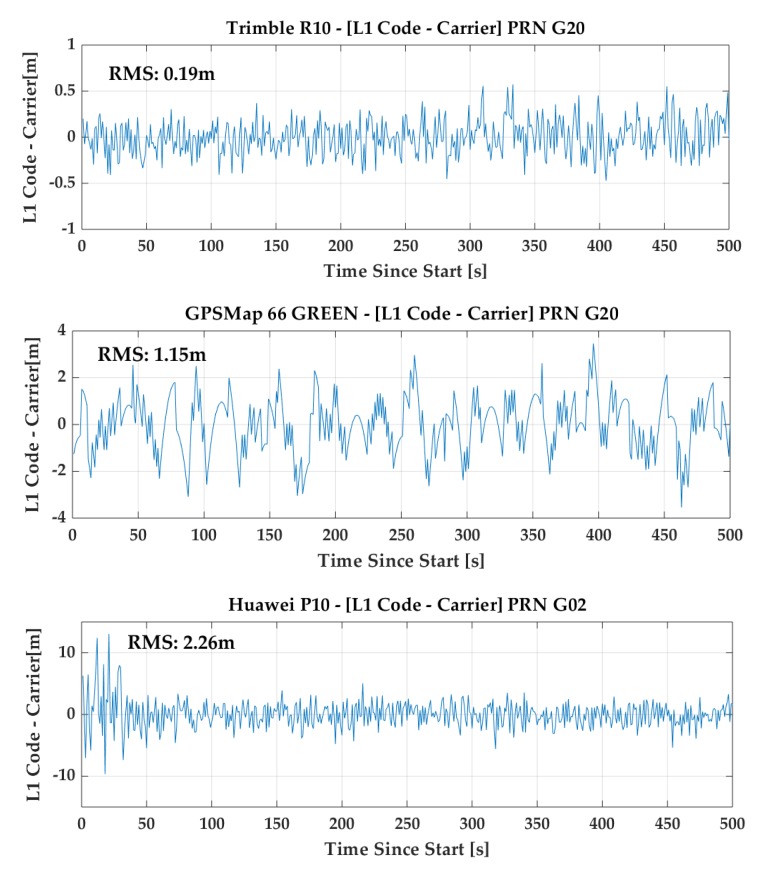
Quality of GPS L1 code measurements of Trimble R10 (**top**), Garmin GPSMap 66 (**middle**) and Huawei P10 (**bottom**) in a low multipath environment using the code-minus-carrier method.

**Figure 3:**
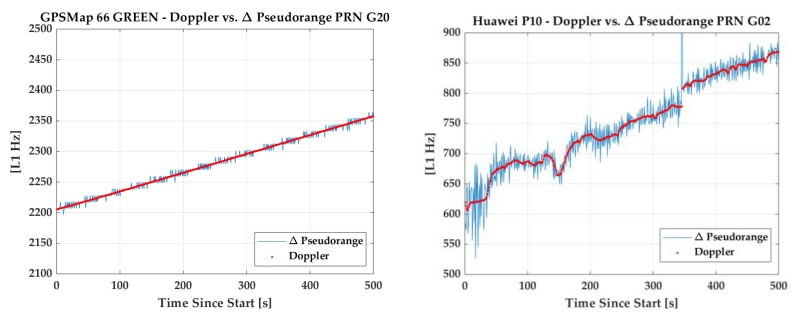
Quality of GPS L1 Doppler and delta ranges of Garmin GPSMap 66 (**left**) and Huawei P10 (**right**) in a low multipath environment.

In order to show the code quality of the units used, differences between code and carrier phase measurements in a low multipath environment are depicted in Figure 2 using the code-minus-carrier approach over periods of 500 s. As expected, the R10 code quality is best, with RMS value of 0.19 m, while corresponding values are 1.15 m and 2.26 m for the GPSMap 66 and P10, respectively. Continuous carrier phase sequences longer than 500 s were rare with the P10 but normal with the GPSMap 66 under clear sky conditions, likely due to Android 8 operating system limitations. The P10 code performance is similar to that of the Huawei Mate 9 tested by [3] and other Android phones (e.g., [4]). The GPSMap 66 performance obtained with Galileo E1 satellites was similar to the above obtained with GPS and no ambiguous E1 code measurement issue arose, unlike the case of some Android phones (ibid). A comparison of instantaneous Doppler and pseudorange differences is shown in Figure 3 for the GPSMap 66 and P10 units, respectively. The P10 data was collected in a moving vehicle.

## 3. Results and Analysis

### 3.1. High Multipath Single Point Static Positioning and Antenna Effect

**Figure 4:**
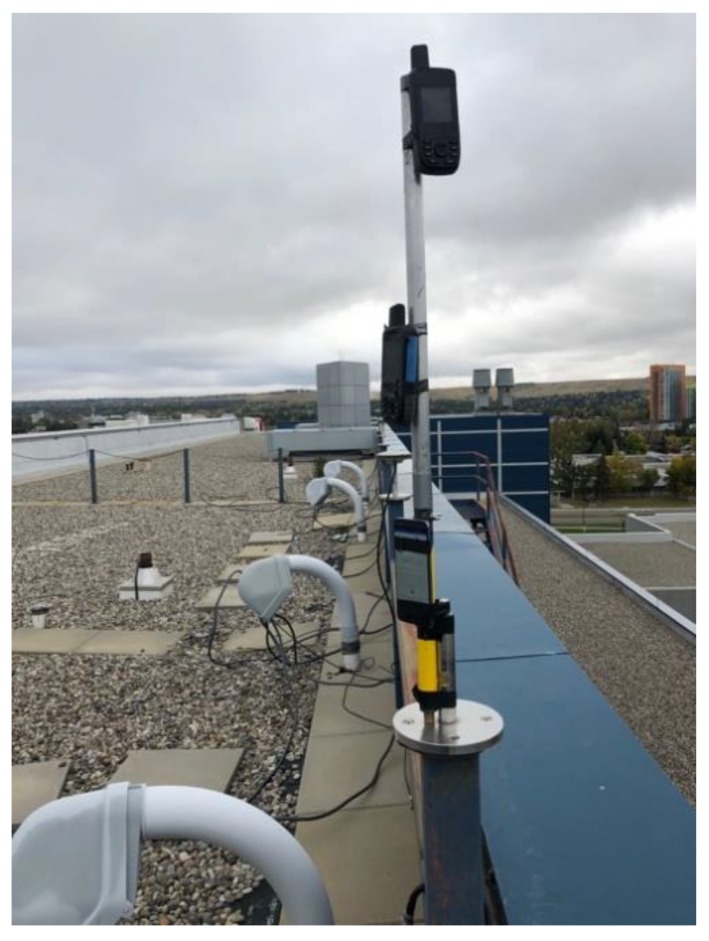
GPSMap 66 and P10 testing in a high multipath environment.

**Figure 5:**
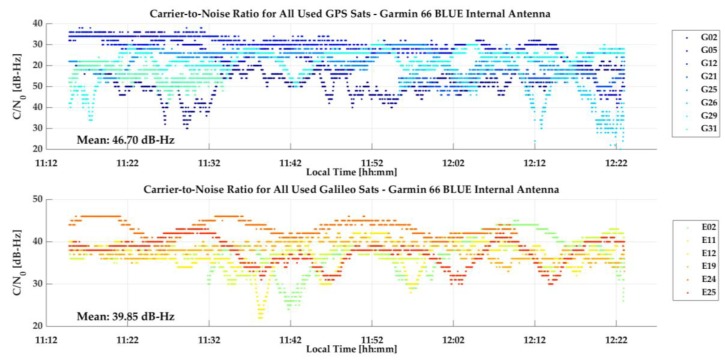
GPSMap 66 blue GPS and Galileo C/N_0_ measurements in the high multipath environment shown in Figure 4.

This test was carried out on the roof top of the CCIT building of the University of Calgary, a high multipath environment. The units were mounted on a vertical pole and separated by 30 cm as shown in Figure 4, with the units’ own antennas, referred to as “internal” antennas in the sequel to distinguish them from the use of an external geodetic antenna as will be reported later. The observation period was selected to have Galileo coverage of up to five SVs with a PDOP of 3 or better. The GPS and Galileo measurements were processed separately in epoch-by-epoch L1 and E1 unfiltered kinematic code mode (using RTKLib). The GPS and Galileo C/N_0_ measurements of the 66 blue unit are shown in Figure 5. Cyclic attenuation of over 20 dB-Hz is characteristic of high specular multipath. Absolute C/N_0_ differences between SVs are generally caused by SV elevation differences. The apparent higher attenuation of the limited Galileo constellation is due to its generally lower SVs elevation than many GPS SVs at this time.

**Figure 6:**
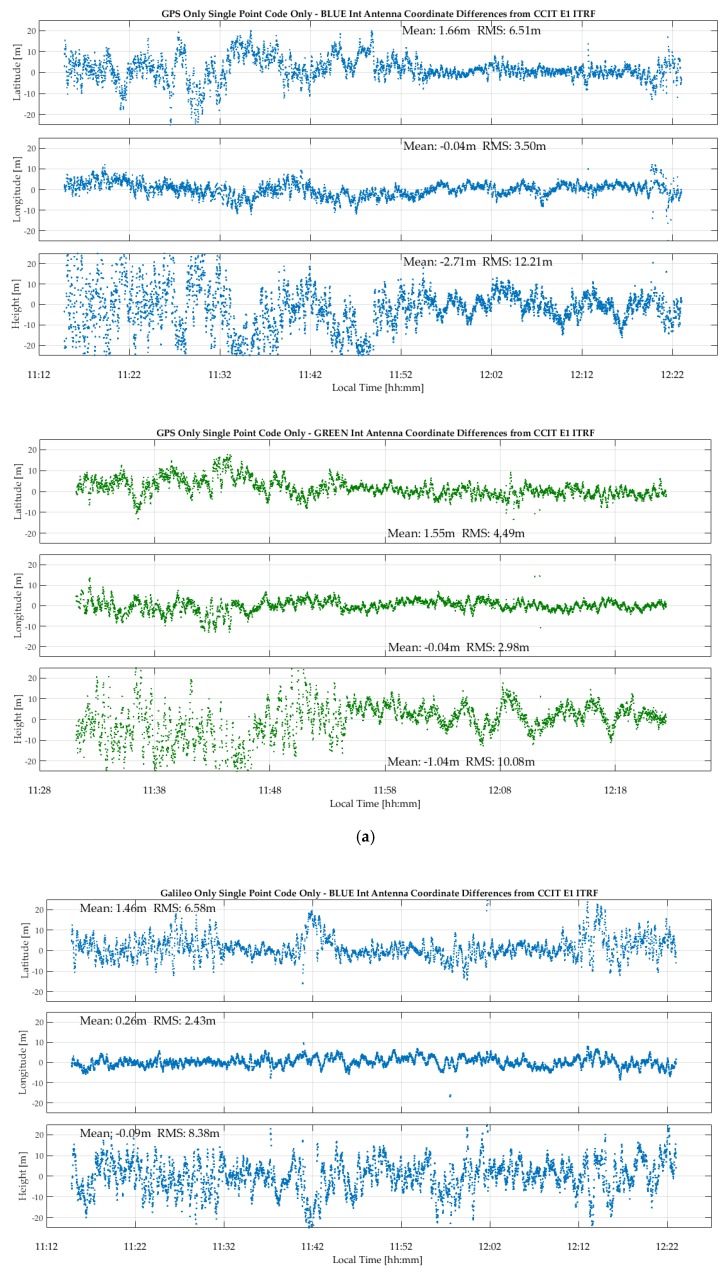

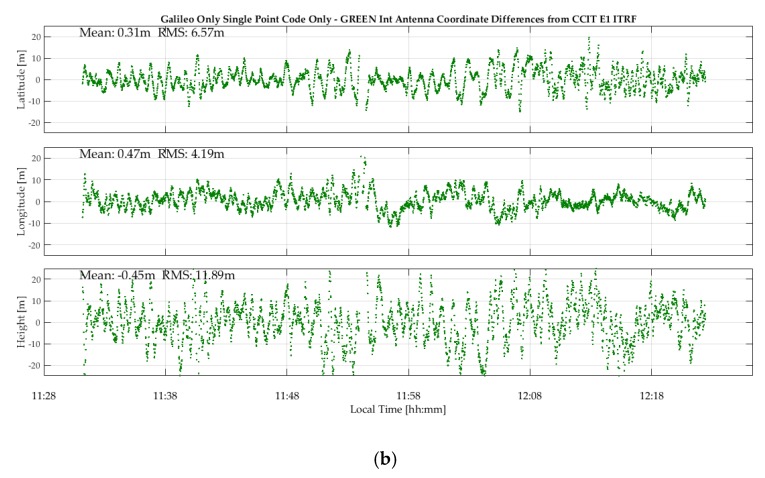
(**a**) GPSMap 66 epoch-by-epoch GPS coordinate variations with respect with ITRF2014 coordinates under high multipath shown in Figure 4. (**b**) GPSMap 66 epoch-by-epoch Galileo coordinate variations with respect with ITRF2014 coordinates under high multipath shown in Figure 4.

The corresponding GPS and Galileo epoch-by-epoch coordinates during the test period are shown in Figure 6a,b, respectively. GPSMap 66 GPS latitude, longitude and height RMS values are 6.5, 3.5 and 12.2 m for the blue unit and 4.5, 3.0 and 10.1 m for the green unit. The higher height RMS values are expected due to normal height observability limitation and high multipath environment. Corresponding Galileo RMS values are 6.6, 2.4 and 8.4 m for the blue unit and 6.6, 4.2 and 11.9 m for the green unit, hence not significantly different from the GPS values. Cyclic coordinate variations are due to multipath as in the case of C/N_0_ measurements. Since the coordinates are based on single-frequency measurements, the effect of the ionosphere would affect the average mean differences. However, given the high noise level, it is not possible to confirm any significant ionospheric effect, even on the height component, the more so during the current period of minimum solar activity. This reference point was observed frequently with the dual-frequency Trimble R10 during the month long period of the tests. The data was processed successively in L1 and L1/L2 mode and the resulting coordinate differences, a direct measure of the effect of the ionosphere on coordinates, were consistently lower than 10 cm. No significant biases are observed between these coordinates and the ITRF 2014 reference coordinates of the observation point.

Since the two GPSMap 66 units were collecting data at the same time, the results, within the noise level, should be the same. However, the units were separated in height by 30 cm and affected by different multipath. This spatial difference is the main reason for the slightly different results between the two units. Despite the limited number of Galileo SVs, the results are not significantly different from GPS results. Given the high multipath, the potential advantage of the higher number of GPS SVs is buried in noise.

**Figure 7:**
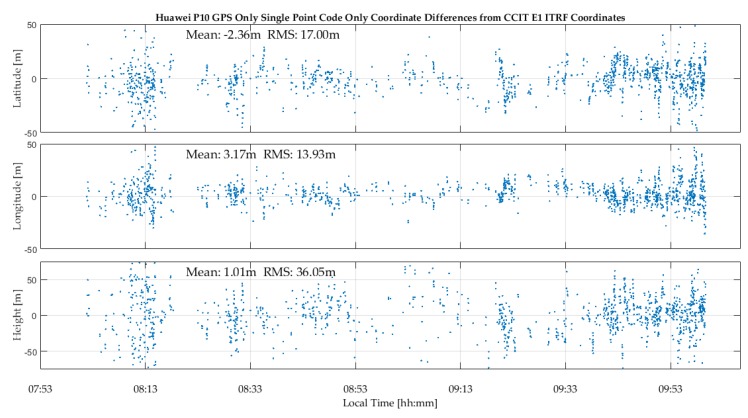
P10 epoch-by-epoch GPS coordinate variations with respect with ITRF2014 coordinates under high multipath shown in Figure 4 using internal antenna.

The P10 results for the above test resulted in discontinuous GPS positions and a limited number of Galileo SVs being tracked. Sub-optimal P10 GNSS data recording during the tests reported in this paper occurred frequently. A previous test, whose GPS results are shown in Figure 7, produced continuous results. In the high multipath environment shown in Figure 4, the P10 antenna is not as effective as the GPSMap 66 to deal with multipath and the resulting position coordinate noise is relatively high.

#### Use of External Geodetic Antenna

**Figure 8:**
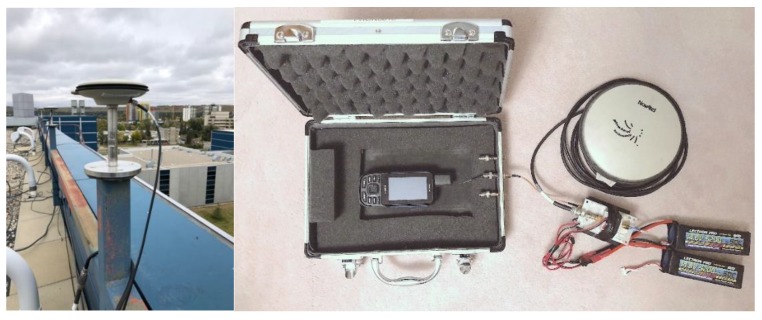
GPSMap 66 testing with external geodetic antenna.

The above test was repeated with one GPSMap 66 unit using an external geodetic antenna, namely the NovAtel 702-GG model (NovAtel Inc, Calgary, AB, Canada) at the same location as shown in Figure 8 (left). This test was done to demonstrate antenna importance in such a high multipath environment. The GPSMap 66 and P10 units do not have connectors for an external antenna. The procedure to use the external antenna in the absence of a wired connection is fully described in [3] during evaluation of the Huawei Mate 9. The 702-GG was connected to a metallic box with its open-ended wire re-transmitting inside where the unit under test locked on the 702-GG signals. The inside of the box was lined with RF absorbent material to further decrease the strength of signals that may be coming directly from the outside through the box walls and slits. The hardware assembly is shown on the right side of Figure 8. The issue of the potential effect of re-radiation inside the box was analysed by [5] and no effect was detected, therefore validating the above procedure.

**Figure 9:**
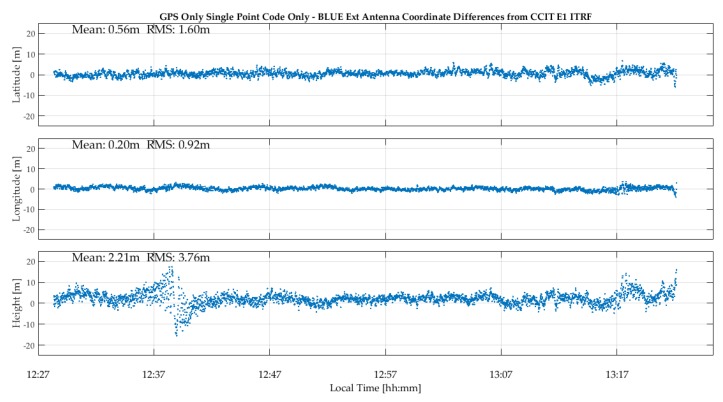

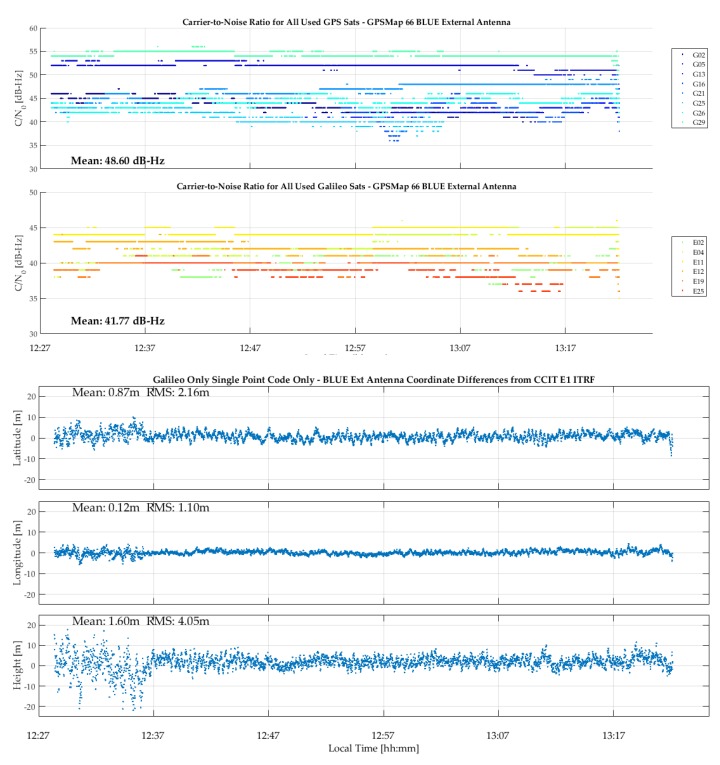
GPSMap 66 C/N_0_ variations and epoch-by-epoch GPS and Galileo coordinate variations using external geodetic antenna.

**Figure 10:**
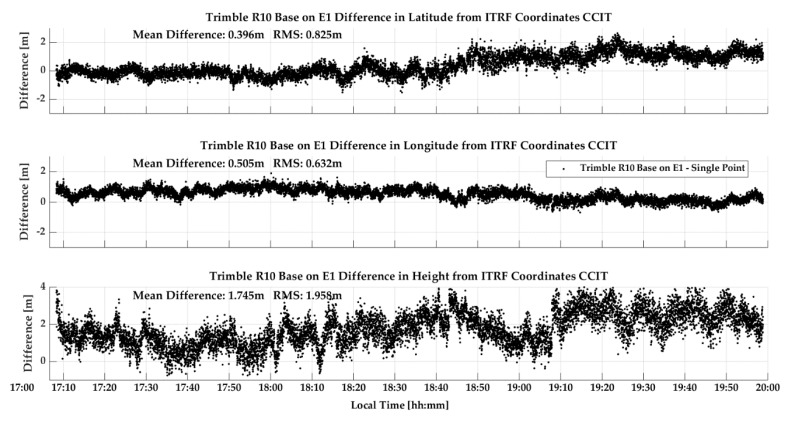
Trimble R10 epoch-by-epoch L1 C/A code coordinate variations in high multipath environment shown in Figure 8.

The test was conducted over a 50-min period during which Galileo coverage was available. The C/N_0_ values and code-based epoch-by-epoch single point positions are shown in Figure 9. The C/N_0_ fluctuations are much smaller than when using the internal antenna due to the better multipath rejection capability of the geodetic antenna. This leads to a higher average gain of 2 dB-Hz with respect to the use of the internal antenna. The temporal coordinate variations are much smaller and demonstrate antenna importance under high multipath conditions. The GPSMap 66 latitude, longitude and height RMS values are now 1.6, 0.9 and 3.8 m for GPS and 2.2, 1.1 and 4.0 m for Galileo, which is two to three times better than the results shown in Figure 6 when using the unit’s internal antenna and only slightly higher than those of the Trimble R10 L1 code epoch-by-epoch values of 0.8, 0.6 and 2.0 m shown in Figure 10 at the same location, which is remarkable. In single point positioning mode, the effect of satellite orbit and clock errors, in addition to those of the atmosphere, limits accuracy achievable, regardless of the receiver used. The use of carrier phase in differential mode leads to different levels of performance between the receivers used as will be demonstrated in the sequel with tests in differential mode.

**Figure 11:**
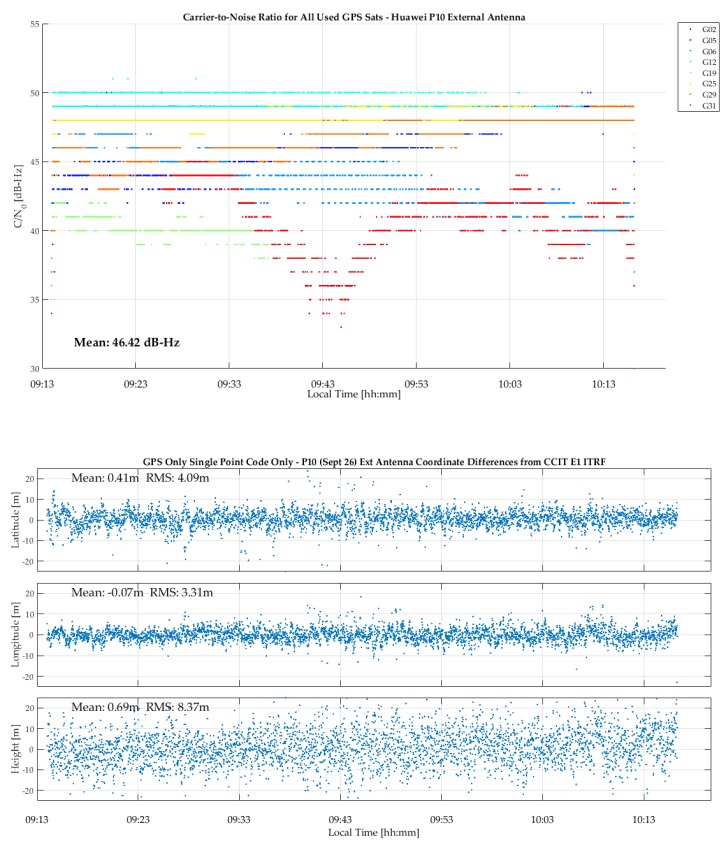
P10 measured C/N_0_ values and epoch-by-epoch GPS coordinate variations with respect with ITRF2014 coordinates under high multipath using external antenna.

P10 position coordinate results in the same high multipath environment with the external antenna are shown in Figure 11. Performance remains substantially poorer than that of the GPSMap 66 shown in Figure 9 but is still better than that shown in Figure 7 for the P10 without the benefit of an external antenna.

### 3.2. Low Multipath Single Point and Differential Positioning

**Figure 12:**
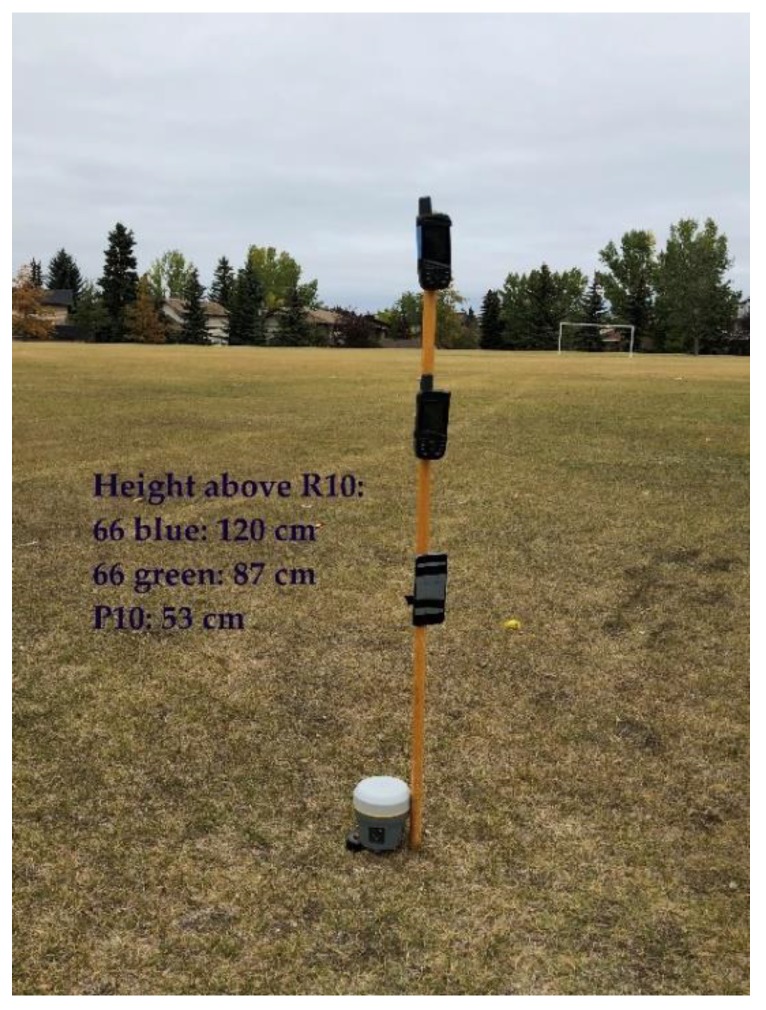
GPSMap 66 and P10 unit testing in low multipath environment. Trimble R10 added to provide accurate reference positions (Coach Hill, 12Sep18).

A lower multipath test was conducted in the middle of a flat playground with no obstruction in a radius of 100 m, a section of which is shown in Figure 12. The two GPSMap 66 and Huawei units being tested were mounted on a vertical wooden pole with the vertical separations shown in the figure. The data collection occurred over a period of 75 min during which four to five Galileo SVs were available with a PDOP of 3 to 7. The corresponding values for GPS were 10 to 11 SVs and a PDOP better than 2. A Trimble R10 was installed at the base of the pole and operating in differential mode with a second R10 unit at a reference station 5 km away at the same point as used previously to provide an accurate position of the test point to evaluate the other units.

**Figure 13:**
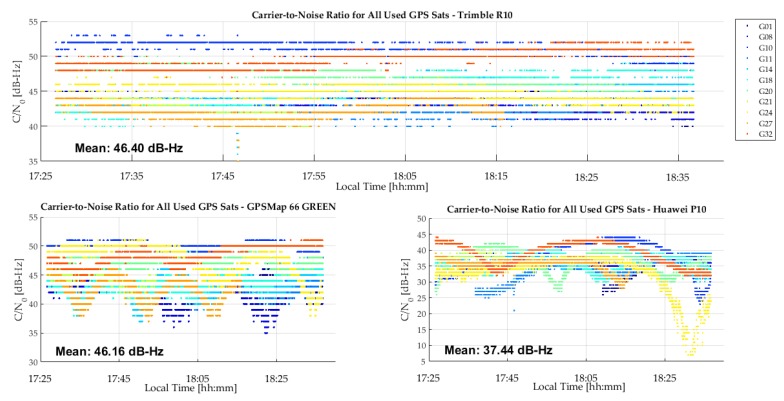
C/N_0_ measurements of R10, GPSMap 66 and P10 during test shown in Figure 12.

The C/N_0_ measurements of the R10, GPSMap 66 green and P10 units are shown in Figure 13. The GPSMap 66 blue unit has similar values. The R10 values are better than those of the GPSMap 66 for a few SVs, although the averages are similar. However, no cyclic variations are present in the R10 values, demonstrating the high multipath rejection capability of its antenna. Although the test environment was considered to be low multipath, cyclic variations likely due to multipath caused by buildings in a radius of 200 m are still present in the GPSMap 66 unit results. Helix antennas, although compact and well suited for hand held units, are not as effective for multipath rejection as high performance but larger geodetic antennas. The P10 C/N_0_ values are much lower, with an average of 8 to 9 dB lower than those of the R10 and GPSMap 66 units. The P10 provided carrier phase for only very short periods, which is not surprising given the low C/N_0_ values. Another test with the GPSMap 66 on a 2200 m mountain summit with the unit on the ground resulted in a C/N_0_ value average of 1 dB-Hz higher than in the current case, due to the absence of any natural or person-made objects below the 360° horizon.

#### 3.2.1. Precise Point Positioning (PPP) Static Results

The distance between the reference station and the test point was 5 km, hence the effect of the ionosphere would have been nearly identical at each point. Three hours of R10 measurements at the reference station made during the test were processed successively in L1/L2 and L1 only PPP mode. The coordinate differences provide as excellent estimate of the effect of the ionosphere during the test. The differences (L1/L2 − L1) were 1, −9 and 13 cm in the latitude, longitude and height components, hence insignificant on the GPSMap 66 L1 results. The R10 L1/L2 and GPSMap 66 L1 measurements at the test point were also processed in PPP static mode. The differences between the R10 and GPSMap 66 blue unit were 43, 39 and −35 cm in the latitude, longitude and height components. The corresponding differences for the GPSMap 66 green unit were 34, −32 and 2 cm. The results demonstrate the capability of the GPSMap 66 for PPP applications at the 1 m RMS or better level under low ionospheric conditions. The P10 data was also submitted to the NRCan PPP service in static mode. The outcome was returned in code only kinematic mode due to the noisy data. No carrier phase nor Galileo data was available on the P10 during the test.

#### 3.2.2. Epoch-by-Epoch Single Point and Differential Mode

**Figure 14:**
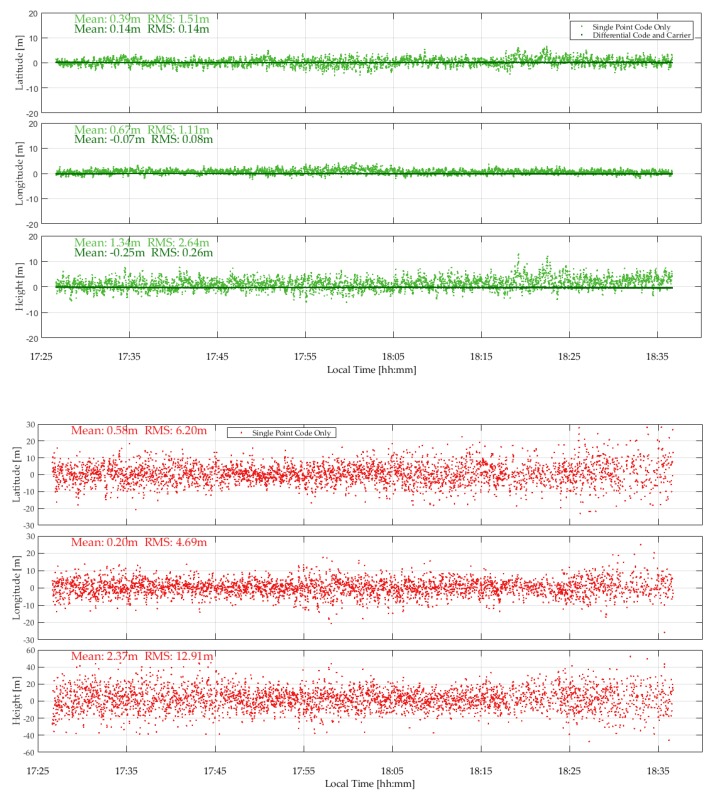
GPSMap 66 and P10 GPS epoch-by-epoch single point and differential code/carrier phase (GPSMap 66 unit only) coordinates variations with respect to reference values (Coach Hill, 13Sep18).

The GPSMap 66 and P10 measurements were successively processed in epoch-by-epoch single point single frequency code mode and differential code/carrier phase (when available) mode using RTKLib and the resulting coordinates were compared to the test point reference values. The results for GPS are shown in the two plots of Figure 14. In single point mode, the superior helix antenna of the GPSMap 66 showed its advantage with RMS values of 1.51, 1.11 and 2.64 m in the latitude, longitude and height components, compared to the corresponding P10 RMS values of 4.20, 4.69 and 12.9 m. The performance of the GPSMap 66 in differential code/carrier phase mode, with RMS values of 14, 8 and 26 cm in latitude, longitude and height, is impressive. The code/carrier phase RTKLib solution was in float mode 100% of the time. Carrier phase lock was maintained more or less continuously.

The P10 unit did not output carrier phase measurements nor Galileo data during the test. The single point GPS results are shown in the second plot of Figure 14. No differential processing was pursued with the P10.

**Figure 15:**
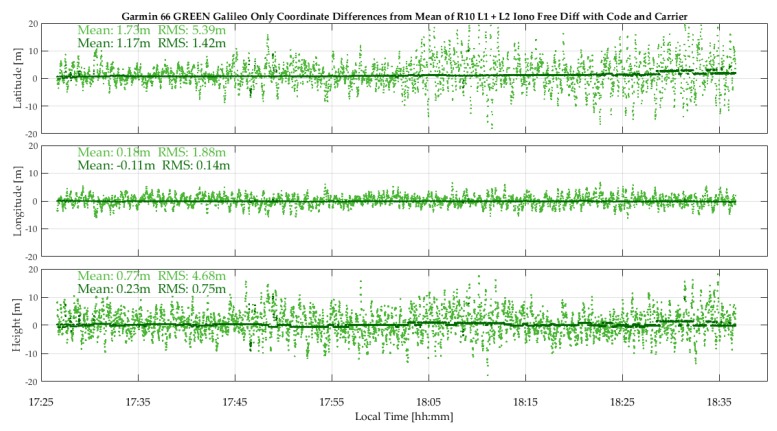
GPSMap 66 Galileo epoch-by-epoch single point and differential code/carrier phase coordinate variations with respect to reference values (Coach Hill, 13Sep18).

The Galileo coverage was sufficient to obtain solutions with the GPSMap 66 units. The measured C/N_0_ values, not shown here, range from 42 to 52 dB-Hz for the R10 and 34 to 46 dB-Hz for the 66 green. The mean difference between the two units is 5 dB-Hz. The Galileo E1 code single point and code/carrier phase position results are shown in Figure 15. The single point RMS values of 5.39, 1.88 and 4.68 m in latitude, longitude and height are predictably higher than the corresponding values reported for GPS, given a PDOP of 4 to 7 for Galileo versus 2.7 for GPS and four to five SVs for Galileo versus 10 to for GPS. The differential code/carrier phase RMS values of 1.42, 0.14 and 0.75 m versus those of GPS are however roughly in the same range, showing a similar level of code and carrier phase performance tracking at the individual signal level within the above error bounds.

### 3.3. Vehicular Positioning

**Figure 16:**
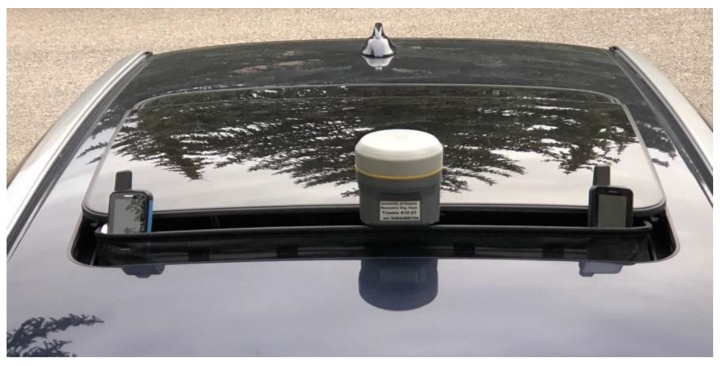
Vehicular test antenna mounting.

**Figure 17:**
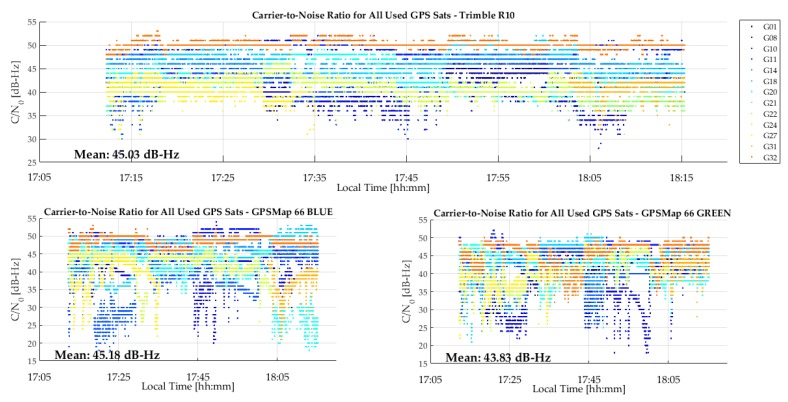
Carrier-to-Noise Ratio (C/N_0_) values of the R10 and 66 units during the vehicular test (22Sep18).

A test to assess the GPSMap 66 units in vehicular mode was carried out on a relatively open 43-km road rectangle at speeds of 80 to 100 km/h. Two 15 km legs of the rectangle was oriented east-west while the two others were north-south. The rectangle was driven twice to assess consistency, resulting in a 2-lap, 86 km trajectory. The R10 and GPSMap 66 units were mounted on a car roof as shown in Figure 16, to secure access to line-of-sight signals. The antennas are at the same height but separated horizontally from each other by 35 cm. There were unavoidable obstructions by foliage and passing vehicles. A R10 unit at a base station 6 to 20 km away was used for differential processing. The differential R10 trajectory served as reference for evaluating the GPSMap 66 trajectories in each coordinate component. The R10 RTKLib code/carrier phase solution was in float mode and its accuracy in each coordinate would have been better than 10 cm much of the time except during short periods when carrier phase lock losses caused by obstructions along the road occurred. Signal attenuation occurring along the trajectory is seen when examining the C/N_0_ measurements of all units in Figure 17. The two GPSMap 66 were 60 cm but still largely exposed to the same attenuation and multipath. Their C/N_0_ patterns are highly correlated, which is also a strong indicator of the high reproducibility of their two helix antenna horizontal and vertical radiation patterns. A code-minus-carrier analysis of the results confirmed that the lower C/N_0_ values were caused by multipath. The plots indicate where trees and other obstructions were present. The attenuation is caused by partial line-of-sight blockage and a mix of specular and diffuse multipath. While the average multipath is the same for all units, that of the GPSMap 66 units exhibits deeper attenuations during short time periods, which would be antenna related.

**Figure 18:**
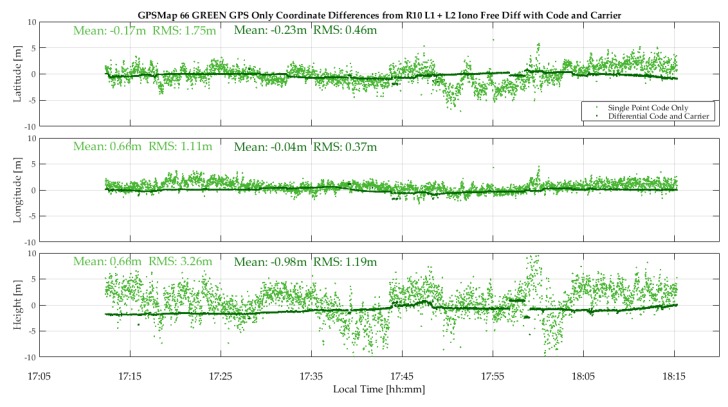

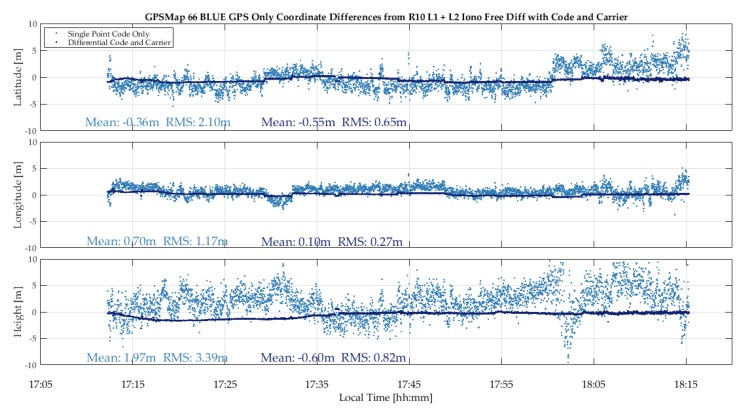
GPS single point and differential (code/carrier phase) agreement of the GPSMap 66 units with the reference trajectory during vehicular test (22Sep18).

The GPSMap 66 code measurements were processed in epoch-by-epoch code single point mode and in differential code/carrier phase mode, the latter over 95% in float carrier phase ambiguity mode. The differences with respect to the R10 reference trajectory are shown in light and dark blue, and light and dark green in the two plots of Figure 18. The single point code position performances of the two units are at the same level, with RMS values of 2.10 m, 1.17 m and 3.39 m in latitude, longitude and height for the blue unit versus 1.75 m, 1.11 m and 3.26 m for the green unit. The mean differences are well within the above RMS values. These differences are due mostly to a combination of GPSMap 66 code multipath and noise. The differential code/carrier phase results are significantly better with RMS values of 0.65 m, 0.27 m and 0.82 m in latitude, longitude and height for the blue unit and 0.46 m, 0.37 m and 1.19 m for the green unit, hence clearly better than the 1 m RMS level. The float ambiguity mode of the GPSMap RTKLib code/carrier phase solution means that phase lock was maintained on a sufficient number of GPS SVs to produce a carrier phase based solution, albeit float, over 95% of the time.

The effects of carrier phase ambiguity float mode solutions with the GPSMap 66 units and the R10 unit some of the time are due to signal shading that results in occasional losses of phase lock. This has a significant impact on the magnitude of the mean and RMS differences reported above. In float mode, code measurements are used with varying weights and their noise and multipath enter the solutions. The ionosphere has no effect on the GPSMap 66 L1 measurements in differential mode. In single point mode, it has no significant effect in this case either. The magnitude of the ionospheric effect was measured by successively processing the R10 data at the nearby base station in L1/L2 and L1 mode. The latitude, longitude and height differences (L1/L2 − L1) were −3, −4 and −4 cm.

**Figure 19:**
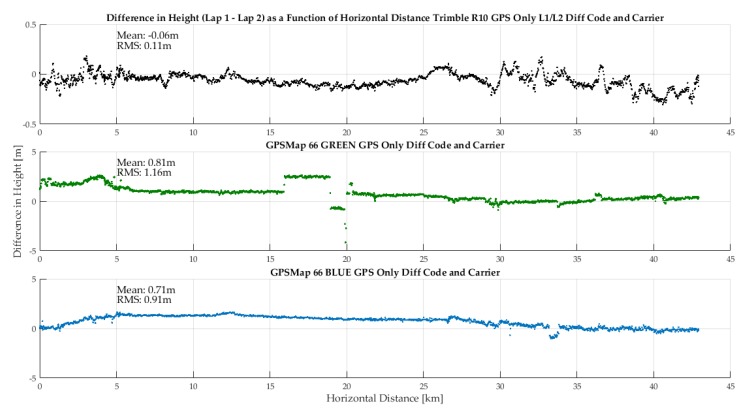
Height repeatability of units between two laps of the same trajectory during the vehicular test (22Sep18).

The two iterations of the rectangular vehicular trajectory (Lap 1 and 2) allows one to assess height profile repeatability as a function of horizontal coordinates. This is done in Figure 19 for the R10 and the two GPSMap 66 units, all in differential code and carrier phase mode. Predictably, the R10 height repeatability is within 10 cm except where obstructions along the road result in losses of phase lock. Repeatability of the GPSMap 66 units is lower, with the RMS difference being slightly over 1 m for the green unit. These results confirm independently the validity of the mean and RMS values shown in Figure 18.

**Figure 20:**
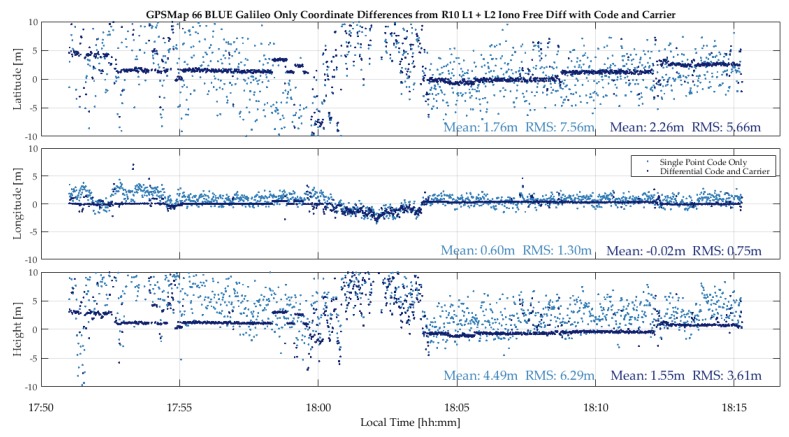
Galileo single point and differential (code/carrier phase) performance of the 66 unit during vehicular test (22Sep18).

Galileo only results: Although the number of satellite available above the horizon was only five and in a sub-optimal geometry, enough Galileo satellites were available to calculate an independent solution for part of the time as shown in Figure 20. Despite the poor geometry and signal shading, the differential code/carrier solution was available some 70% of the time during the 25-minperiod. The single point position is noisier than the GPS one due to higher DOP values and lower measurement redundancy.

**Figure 21:**
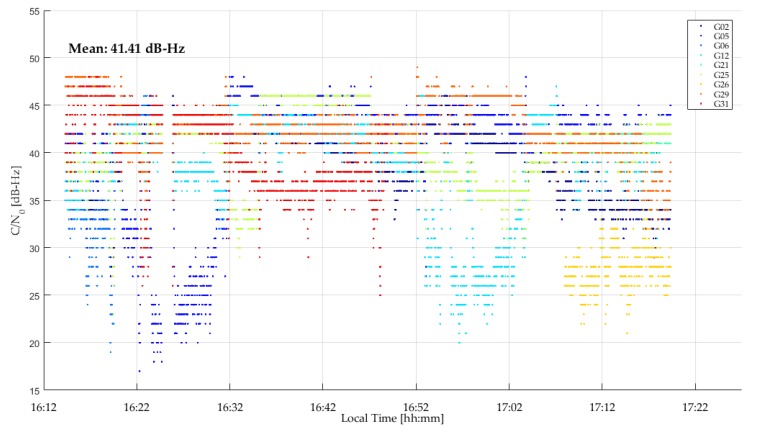
GPS Carrier-to-Noise Ratio (C/N_0_) values of the P10 during vehicular test (28Jun18).

**Figure 22:**
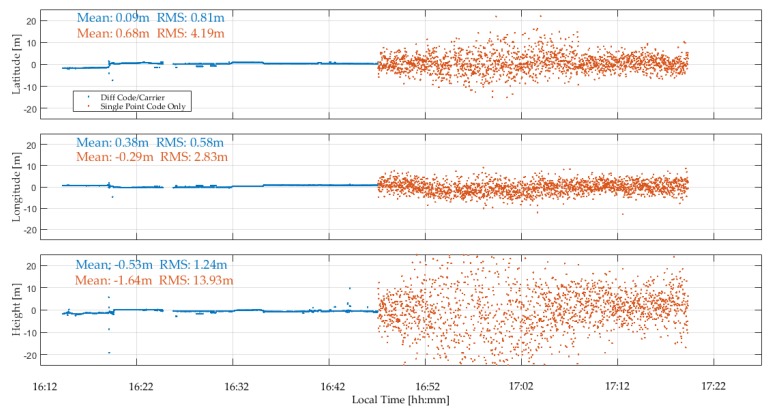
GPS single point and differential (code/carrier phase and code only) accuracy of P10 during vehicular test (28Jun18).

P10 Vehicular test: The P10 was tested in the same manner as above but at a different time on the same trajectory, again with two laps. The GPS C/N_0_ values, shown in Figure 21, exhibit the same attenuation pattern as seen for the GPSMap 66 test, although the average C/N_0_ is lower by 4 dB-Hz, a highly significant and detrimental effect on carrier phase tracking. The comparison of the P10 and R10 reference trajectory is shown in Figure 22 During the first lap, the P10 maintained phase lock and the results are very good and similar to those of the GPSMap 66 in the previous test. During the second lap, phase lock was lost and did not recover, likely due to Android 8 operating system limitations as mentioned earlier. Hence the second half of Figure 22 shows performance in differential code mode only. Galileo coverage was available and the P10 was able to successfully track signals during various periods but only in E1 code mode. The geometry of the SVs tracked was not sufficient to calculate reliable positions.

### 3.4. Indoor Testing

In order to study GNSS signal behavior with the GPSMap 66 and P10 units in an indoor environment, a test was conducted in the basement of a private residence. GPSMap 66 results are reported below. The P10 only recorded a few GPS SVs periodically, although it was initialized outside, hence there are no results to report. Indoor environments are difficult to fully characterize in term of GNSS signal behavior. An important metric is signal attenuation. Others are the size of the room in which the test occurs, wall materials and windows as these impact multipath characteristics. The 90-min test reported herein took place when up to six Galileo SVs were available. The room size was 4 m × 4 m × 2.5 m, concrete walls were 1 m above the ground and one outer wall had a 2.5 m × 1 m window. The coordinates were transferred from an outdoor position with accuracy of 1 m.

**Figure 23:**
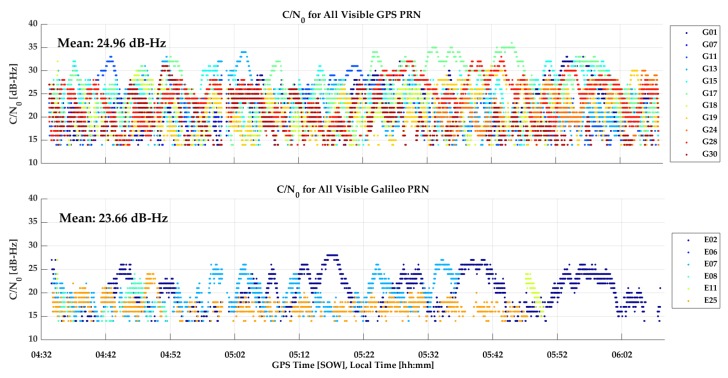
GPSMap 66 GPS and Galileo C/N_0_ values during indoor test (18Sep18).

**Figure 24:**
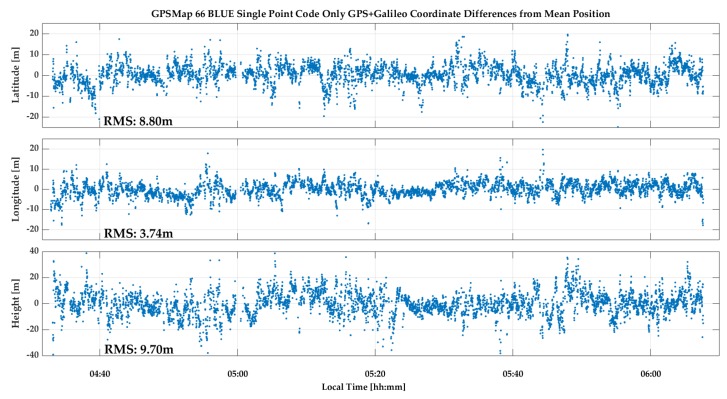
GPSMap 66 Combined GPS/Galileo epoch-by-epoch single point positions during indoor test.

**Figure 25:**
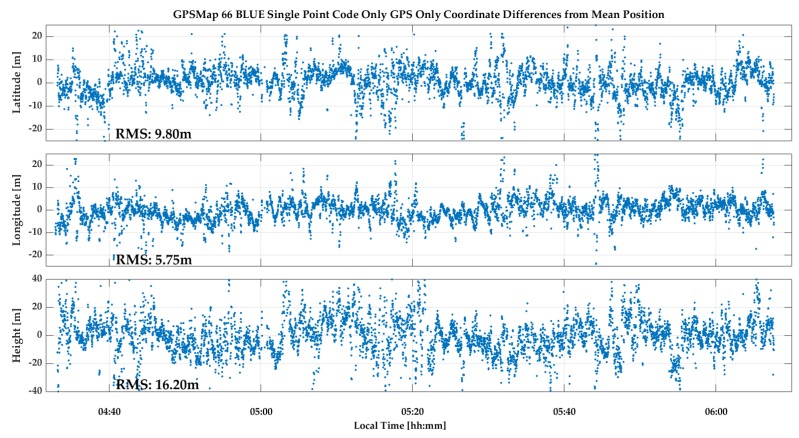
GPSMap 66 epoch-by-epoch GPS single point positions during indoor test.

**Figure 26:**
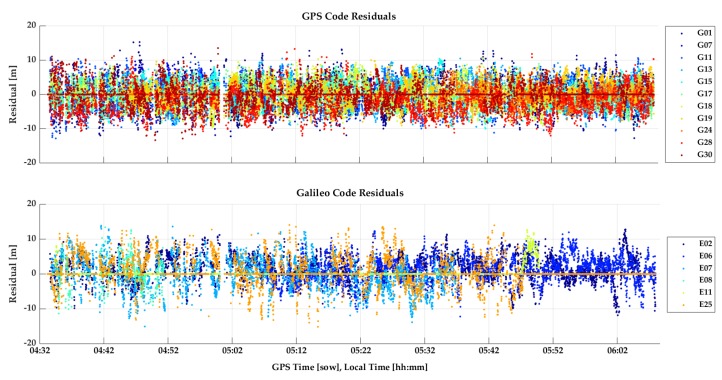
Combined GPSMap 66 GPS and Galileo residuals from least-squares solution of indoor test.

The GPSMap 66 C/N_0_ values measured during the test are shown in Figure 23. All values are below 35 dB-Hz and drop to below 15 dB-Hz. The unit was able to acquire and track many signals for continuous periods of time. The cyclic variations are typical of multipath. The lower Galileo signal values are due to generally lower SV elevations. Again, code measurements were processed in epoch-by-epoch single point mode to evaluate their stability. The combined GPS/Galileo and GPS only solutions are shown in Figure 24 and Figure 25. The RMS coordinate variations are 8.8, 3.7 and 9.7 m in latitude, longitude and height for the combined solution, and 9.8, 5.8 and 16.2 m for GPS only. The GPS only results are slightly poorer, demonstrating the importance of using as many signals as possible (GPS + Galileo in this case) to somewhat mitigate noise and multipath in such a harsh environment. The GPS/Galileo average coordinates agree at the level of 1.5 m with the values transferred from the outdoors. The residuals of the epoch-by-epoch least-squares solutions are shown in Figure 26 and are within a band of 20 m.

### 3.5. Urban Canyon Signal Behavior Study

**Figure 27:**
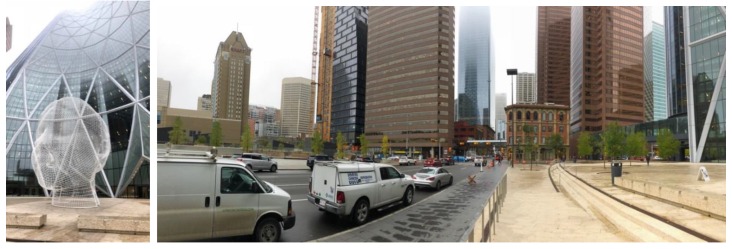
GPSMap 66 was located inside sculpture in front of The Bow (**left**). South and west skyline left of sculpture (**right**).

**Figure 28:**
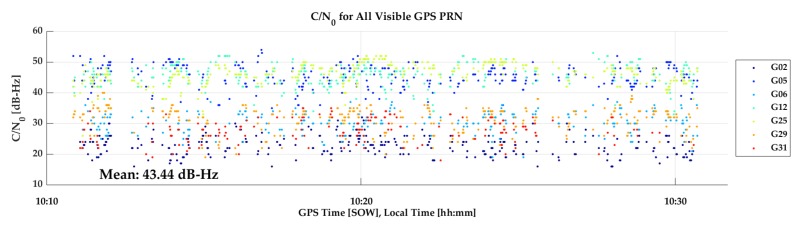
GPSMap 66 GPS C/N_0_ values measured inside sculpture shown in Figure 29.

**Figure 29:**
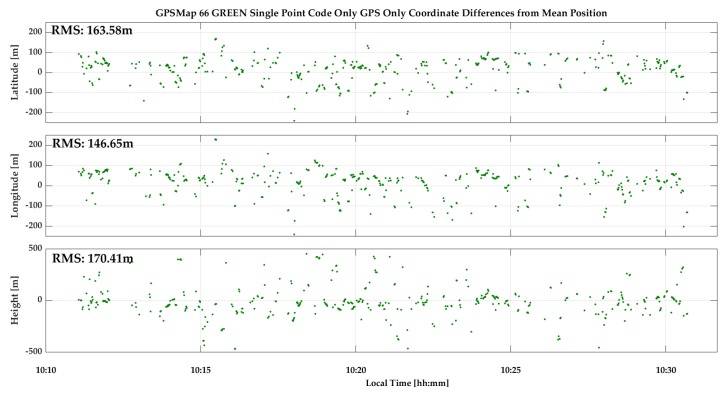
GPS epoch-by-epoch coordinate variations inside sculpture shown in Figure 27.

In order to study GNSS signal behavior under harsh urban conditions, a 20-min test was conducted inside the 12-m high metallic mesh sculpture located in front of the 235-m high The Bow building in Calgary. The sculpture is shown on the left side of Figure 27 while the south and west skyline view from the sculpture is shown in the panoramic picture on the right. The skyline to the east is somewhat more open. As a consequence, line-of-sight signals are limited and all signals are subject to extreme multipath and attenuation. A GPSMap 66 was held 30-cm above the head of the observer standing inside the sculpture. Sufficient GPS measurements were made to evaluate their behavior. Their C/N_0_ values and coordinate variations are shown in Figure 28 and Figure 29. The C/N_0_ spread ranges from 18 to 51 dB-Hz. Since this is an open area, multipath delays are longer, resulting in RMS coordinate component variations of the order of 150 m.

## 4. Conclusions

The realistic static and kinematic tests conducted in this study with the robust hand held GNSS GPSMap 66 unit capable of recording GNSS raw code, carrier phase and Doppler measurements demonstrate its capability under a variety of conditions. The benefit of its helix antenna over that of a smartphone leads to better multipath rejection and generally continuous carrier phase measurements under line-of-sight conditions. The Huawei P10 (operating with the Android 8 operating system) carrier phase measurement continuity was poor in most instances. The 7 to 8 dB-Hz gain advantage of the GPSMap 66 over the P10 is one reason for its relative better performance. The differential positioning accuracy performance of the GPSMap 66 in vehicular mode was slightly better than 1 m RMS in each of the three coordinate components. The use of an external geodetic antenna in a high multipath environment demonstrated a further advantage over a helix antenna through reduction of multipath and signal attenuation, and better single point positioning. In an indoor environment with C/N_0_ values of 15 to 35 dB-Hz, the GPSMap 66 was able to acquire and track sufficient signals to maintain one sigma accuracy of the order of 10 m RMS in each coordinate component. Although Galileo coverage is still limited, the unit produced Galileo only positions that were at the same general level of performance as that of GPS. The PPP approach was able to deliver sub-metre accuracy in each of the coordinates using 75-min data sets. The level of performance obtained in this study is similar to that obtained with the Garmin Rino 750 as described in [6]. Raw GNSS data with this unit is not however commercially available. Another analysis reported in [7] using GNSS watches with custom software to provide raw data show results close to those of the GPSMap 66 but under more ideal field conditions.
